# Magnesium intake and vascular structure and function: the Hoorn Study

**DOI:** 10.1007/s00394-021-02667-0

**Published:** 2021-09-07

**Authors:** Emma A. Vermeulen, Hanne B. T. de Jong, Alexander G. A. Blomjous, Coby Eelderink, Tiny Hoekstra, Petra J. M. Elders, Martin H. de Borst, Marc G. Vervloet, Adriana J. van Ballegooijen, Joline W. Beulens

**Affiliations:** 1 Department of Nephrology, Amsterdam UMC, Location VUmc, Amsterdam, The Netherlands; 2 Division of Human Nutrition and Health, Wageningen University and Research, Wageningen, The Netherlands; 3 VUmc Medical Faculty, Amsterdam, The Netherlands; 4 Division of Nephrology, Department of Internal Medicine, University Medical Center Groningen, University of Groningen, Groningen, The Netherlands; 5 Department of General Practice and Elderly Care Medicine, Amsterdam Public Health Research Institute, Amsterdam UMC, Location VUmc, Amsterdam, The Netherlands; 6 Department of Epidemiology and Biostatistics, Amsterdam Public Health Institute, Amsterdam UMC, Location VUmc, De Boelelaan 1117, 1081 HV Amsterdam, The Netherlands

**Keywords:** Magnesium intake, Intima-media thickness, Flow-mediated dilatation, Pulse wave velocity, Augmentation index, Peripheral arterial disease

## Abstract

**Purpose:**

Circulating and dietary magnesium have been shown to be inversely associated with the prevalence of cardiovascular disease (CVD) and mortality in both high and low-risk populations. We aimed to examine the association between dietary magnesium intake and several measures of vascular structure and function in a prospective cohort.

**Methods:**

We included 789 participants who participated in the vascular screening sub-cohort of the Hoorn Study, a population-based, prospective cohort study. Baseline dietary magnesium intake was estimated with a validated food frequency questionnaire and categorised in energy-adjusted magnesium intake tertiles. Several measurements of vascular structure and function were performed at baseline and most measurements were repeated after 8 years of follow-up (*n* = 432). Multivariable linear and logistic regression was performed to study the cross-sectional and longitudinal associations of magnesium intake and intima-media thickness (IMT), augmentation index (Aix), pulse wave velocity (PWV), flow-mediated dilatation (FMD), and peripheral arterial disease (PAD).

**Results:**

Mean absolute magnesium intake was 328 ± 83 mg/day and prior CVD and DM2 was present in 55 and 41% of the participants, respectively. Multivariable regression analyses did not demonstrate associations between magnesium intake and any of the vascular outcomes. Participants in the highest compared to the lowest magnesium intake tertile demonstrated in fully adjusted cross-sectional analyses a PWV of −0.21 m/s (95% confidence interval −1.95, 1.52), a FMD of −0.03% (−0.89, 0.83) and in longitudinal analyses an IMT of 0.01 mm (−0.03, 0.06), an Aix of 0.70% (−1.69, 3.07) and an odds ratio of 0.84 (0.23, 3.11) for PAD

**Conclusion:**

We did not find associations between dietary magnesium intake and multiple markers of vascular structure and function, in either cross-sectional or longitudinal analyses.

**Supplementary Information:**

The online version contains supplementary material available at 10.1007/s00394-021-02667-0.

## Introduction

Cardiovascular disease (CVD) is one of the most common morbidities and the leading cause of death worldwide [1, 2]. In addition to traditional risk factors of CVD, such as physical inactivity, tobacco use, hypertension, hypercholesterolemia, obesity and diabetes, magnesium deficiency gained interest during recent years as a potentially modifiable risk factor of CVD [3–8].

As one of the most abundant intracellular cations, magnesium is required for normal cell physiology and is essential for neuromuscular and cardiovascular function [9]. Several systematic reviews and meta-analyses demonstrated an association between high serum or dietary magnesium and a reduced risk of all-cause and cardiovascular mortality, CVD including coronary heart disease and ischemic stroke, hypertension, and type 2 diabetes (DM2) within the general population [3–7, 10]. Part of this association might be explained by the protective effects of magnesium on vascular calcification, vascular tone, endothelial cell function and low-grade inflammation, influencing both vascular structure and function [9, 11]. The calcification inhibiting effects of magnesium have been demonstrated in animal studies as well [12–15]. Literature on the association between magnesium intake, and markers of vascular structure and function in humans is very limited, while many studies do report on the associations with serum magnesium concentration [16–22], or the effects of magnesium supplementation [23–28]. Yet, besides magnesium concentration and magnesium supplementation, the role of magnesium intake from dietary sources (among others leafy vegetables, nuts, whole grains and seeds) could be of importance in the prevention of vascular impairment on a public health level and for dietary guidelines. Only a few studies investigated the association between dietary magnesium intake and intima-media thickness (IMT) or pulse wave velocity (PWV) [29–32]. However, these studies did not observe consistent associations and were mostly performed cross-sectionally in high-risk populations such as DM2 or chronic kidney disease cohorts. No studies reported on magnesium intake in relation to flow-mediated dilatation (FMD), augmentation index (Aix) or peripheral artery disease (PAD). These vascular markers have been measured in a sub-cohort of the Hoorn study, a population-based prospective cohort in the Netherlands that was initiated to study the prevalence and risk factors of impaired glucose metabolism and DM2.

Therefore, the aim of this study was to examine whether dietary magnesium intake is associated with vascular markers IMT, FMD, PWV, Aix and PAD, cross-sectionally and after 8 years of follow-up in individuals of the vascular screening sub-cohort of the Hoorn study.

## Methods

### Study population

**Fig. 1:**
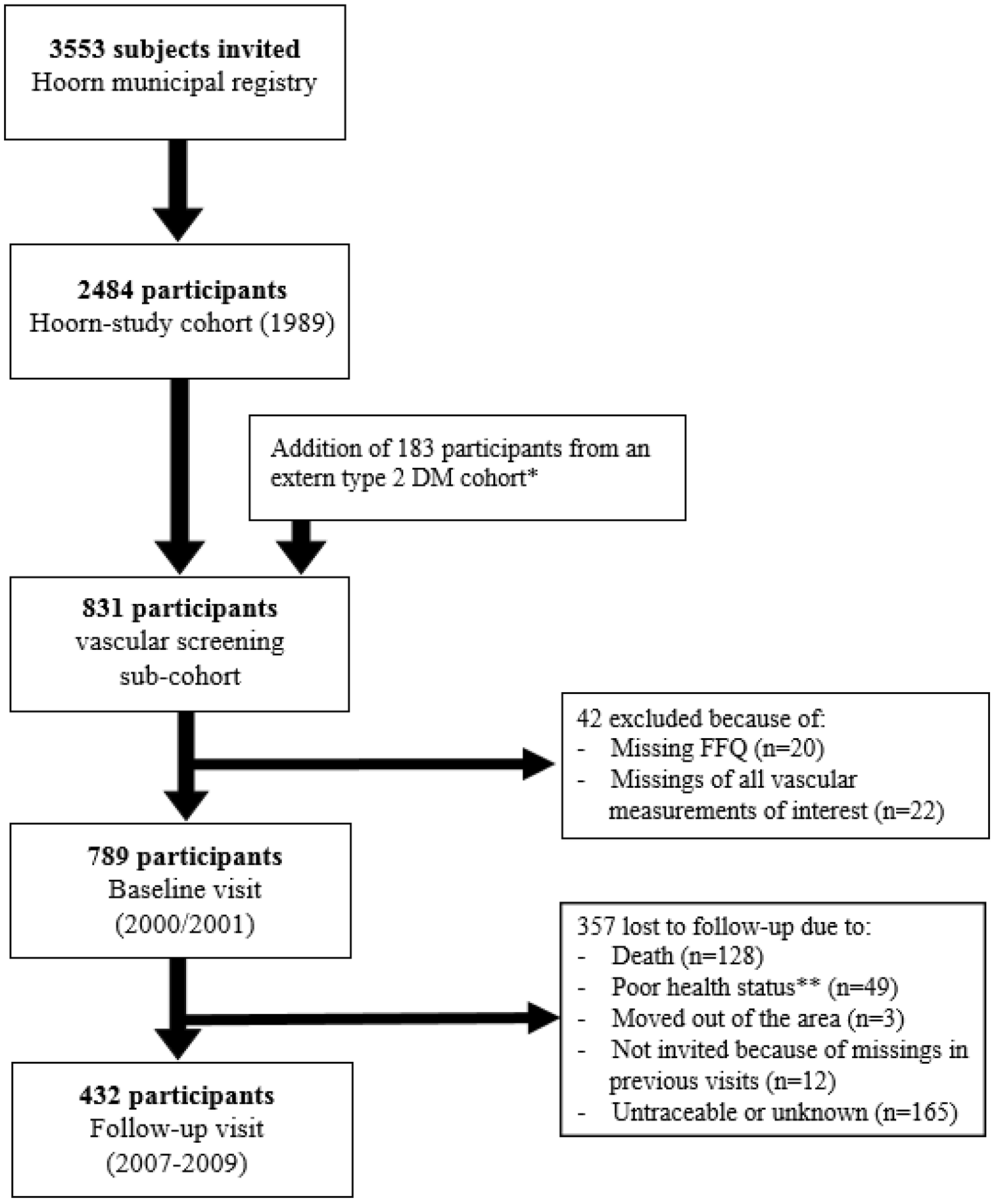
Flow chart of study population. *DM* diabetes mellitus, *FFQ* food frequency questionnaire. *Ref: Spijkerman AM, et al. Diabetes Care. 2002 [34]. **Poor health status included illness, reduced mobility, high age and dementia

The Hoorn Study is a prospective, population-based cohort of Dutch citizens, initiated in 1989 in the Hoorn region. This cohort has been described in detail previously [33]. In brief, 3553 Caucasian men and women, aged between 50 to 75 years, were randomly selected from the municipal registry of the city of Hoorn, resulting in 2484 participants that provided informed consent. For the present study, we used data of a vascular screening sub-cohort (*n* = 831) with additional questionnaires and several measurements of vascular structure and function in 2000–2001, which was considered baseline. This sub-cohort was oversampled for individuals with DM2 and impaired glucose metabolism (IGM) to investigate effect modification by glucose metabolism status [33, 34]. Participants with a missing food frequency questionnaire (FFQ) (*n* = 20) and without any available vascular measurements of interest (*n* = 22) at baseline were excluded from the current analyses, resulting in 789 participants (Fig. 1). Follow-up visits of this vascular screening sub-cohort were performed in 2007–2009 in 432 participants, with repeated measurement of at least one of the vascular measurements. Absence of follow-up visit was due to death (*n* = 128), inability to participate due to poor health status or relocation (*n* = 52), too many missings in previous visit (*n* = 12) and untraceable or unknown reason (*n* = 165). The study was approved by the Ethics Committee of the VU University Medical Centre (Amsterdam, the Netherlands).

### Magnesium intake assessment

Magnesium intake at baseline was assessed based on a validated, 178 item food frequency questionnaire (FFQ), estimating specific food consumption over the previous year. This FFQ demonstrated correlation coefficients of  ≥ 0.61 for fibre and macronutrients and 0.32–0.78 for specific food groups high in magnesium content (bread, cereals, vegetables, nuts and seeds), in comparison to multiple 24 h dietary recalls [35, 36]. Participants filled out the FFQ at home and these questionnaires were checked for completeness after return. Nutrient intake, including magnesium and dietary fibre intake, was calculated by multiplying the amount and frequency of consumption of each food item by its nutrient content using the Dutch Food Composition Table (NEVO) 1993 [37]. Finally, for each nutrient, the total intake was summed for all food items, resulting in a mean nutrient specific, daily intake. No data on supplement use was available, therefore, magnesium intake is defined as solely dietary intake of magnesium.

#### Measurements of vascular structure and function

At baseline, IMT, Aix, PWV, FMD and ankle-brachial index (ABI) were measured by a trained researcher. At the follow-up visit, measurements of IMT, ABI and Aix were repeated. A short description of each of the five markers of vascular structure and function is listed below, more detailed descriptions of each vascular measure are published previously [38–45]. Measurements of the IMT were performed at the location of the common carotid artery using an ultrasound device and we applied the mean of multiple measurements within the analyses [38]. The FMD was assessed through ultrasound examination of the diameter of the brachial artery before and after pressure cuff inflation [39]. FMD is expressed as a percentage and calculated as the mean of three measurements of the maximum diameter after pressure cuff inflation minus the baseline diameter, divided by the baseline diameter. Measurements of both Aix and PWV were performed using the Sphygmocor, AtCor Medical device, measuring the radial artery, and carotid and femoral arteries, respectively [40, 41]. The Aix is expressed as a percentage and calculated as augmentation pressure divided by the tonometrically derived pulse pressure. The carotid-femoral travelled distance was estimated using body height, conform the formula proposed by Weber et al. (body height/4 + 7.28) [42, 43]. Subsequently, the carotid-femoral PWV was calculated as travelled distance in meters divided by transit time in seconds. The ABI was calculated using Doppler-assisted systolic blood pressure measurements at both sides [44, 45]. The clinical cut off of the left or right ABI to classify as a peripheral arterial disease (PAD) was a value below 0.9 [46].

#### Other determinants

Hypertension is defined as systolic blood pressure  ≥ 140 mmHg, diastolic blood pressure  ≥ 90 mmHg or the use of antihypertensive medication. Glucose status is classified according to the WHO criteria (1999) of normal glucose metabolism (NGM), IGM and DM2 [47]. Information on prior CVD was extracted through medical records in combination with the Rose Angina Questionnaire [48]. Subjects filled out multiple questionnaires to obtain information about education level (low, middle, high), smoking status (never, former, current), self-reported physical activity (hours/week), dietary intake (FFQ) and alcohol consumption (no consumption, 0–13 glasses per week,  ≥ 14 glasses per week) [33, 35]. All laboratory values were acquired after overnight fasting and included glucose, haemoglobin A1c (HbA1c), total cholesterol, low-density lipoprotein cholesterol (LDL-cholesterol), high-density lipoprotein cholesterol (HDL-cholesterol), triglycerides, C-reactive protein (CRP), and serum creatinine (Jaffe method). Estimated glomerular filtration rate (eGFR) was calculated according to the chronic kidney disease epidemiology collaboration (CKD-EPI 2009) [49].

### Statistical analyses

We adjusted all dietary intake variables for energy intake using the residual method [50]. Due to differences in total energy intake and sex-specific magnesium intake recommendations, we classified participants into sex-specific tertiles according to their energy-adjusted magnesium intake. The classification into tertiles was separately performed for the cross-sectional (full-cohort) and longitudinal analyses sample (only including those with follow-up measurements). Baseline characteristics are displayed for the magnesium intake tertiles. Variables are displayed as mean and standard deviation when normally distributed, as median and interquartile range [IQR] when the distribution was skewed and for categorical variables as percentage. To examine trends across the magnesium intake tertiles, we performed linear regression analyses or chi-square (Mantel–Haenszel test) for continuous and categorical variables respectively, with magnesium tertiles as a continuous determinant. For this purpose, we first performed a log-transformation for those continuous variables with a right-skewed distribution.

We performed multiple linear regression analyses to investigate the associations between magnesium intake tertiles and IMT, FMD, PWV and Aix, and logistic regression for the association with PAD. The lowest magnesium intake tertile was considered as the reference category. IMT, Aix and PAD were analysed prospectively as well, by adding time and the baseline value as an independent variable to the regression models. In the longitudinal analysis of PAD, cases with PAD at baseline were excluded (*n* = 7). Both PWV and FMD were analysed cross-sectionally only, since these measurements were not repeated during the follow-up visit.

We defined three a priori nested models to adjust for potential confounding in all analyses. The first model is adjusted for age, sex and glucose metabolism status. The second model is additionally adjusted for prior CVD, smoking status and systolic blood pressure. The third model also included energy intake and energy-adjusted fibre intake. Effect modification by age (above or below the median age of 68), glucose status (NGM, IGM or DM2) and prior CVD (yes or no) was tested by adding interaction terms to the regression models with magnesium as a continuous variable. Stratified analyses were performed when significant interaction terms were found (*p* < 0.10) and confirmed with interaction terms for the magnesium intake tertiles.

For the 32 participants with missing data on covariates in the above specified regression models (with a maximum of 3% missing for each covariate), we imputed missing data using multiple imputation with predictive mean matching (10 sets of 10 iterations each). Missing covariates at baseline were CVD (*n* = 23), glucose metabolism status (*n* = 2), smoking (*n* = 5), systolic blood pressure (*n* = 2), glucose status (*n* = 2). We imputed separately for cross-sectional and longitudinal data sets. For the longitudinal analyses, missing data of outcome variables at follow-up (IMT *n* = 11, Aix *n* = 29, ABI *n* = 6) were imputed, however, we did not impute missing data in outcome variables at baseline (up to 19% missing). Therefore, those cases were not included in the longitudinal analyses. We checked imputation of continuous variables by visual inspection of the convergence plots. Sensitivity analyses were performed for complete case analysis and absolute intake tertiles variables, based on the absolute instead of energy-adjusted magnesium intake.

We performed all analyses using IBM SPSS Statistics 24.0 (IBM Corp., Armonk, NY). Two-sided *p* values  < 0.05 were considered statistically significant.

## Results

### Participant characteristics

Participant characteristics at baseline are presented according to sex-specific and energy-adjusted magnesium intake tertiles (Table 1). The mean age was 68.5 ± 7.2 years and 398 (51%) of the participants were men. DM2 and IGM was present in 41% and 23% of the participants, respectively, and 55% of the participants had a history of CVD. Mean absolute magnesium intake was 328 ± 83 mg/day, with a mean of 353 ± 87 mg/day for men and 303 ± 70 mg/day for women. Participants with lower magnesium intake were older, suffered more frequently from CVD, were more frequent smokers, less physically active, had higher triglycerides, higher CRP, lower eGFR, were more frequently proton pump inhibitor users, and had a lower fibre, calcium and phosphate intake. The mean of vascular measurements at baseline are presented in Table 1. In the case of IMT, Aix and PWV, a higher value represents worse vascular structure or function, while a higher FMD reflects better endothelial function.

**Table 1:** Baseline characteristics of the vascular screening sub-cohort of the Hoorn study according to sex-specific and energy-adjusted magnesium intake tertiles (*n* = 789)

	Total cohort	Magnesium intake tertiles		
		Tertile 1	Tertile 2	Tertile 3
*N*	789	262	264	263
Median magnesium intake, mg/day	328	282	328	371
Range of magnesium intake (min–max), mg/day	136–530	136–313	303–350	345–530
Demographics				
Age, years*	68.5 ± 7.2	69.2 ± 7.4	68.9 ± 7.4	67.3 ± 6.7
Male, *n* (%)	404 (51)	133 (51)	134 (51)	134 (51)
Education level, *n* (%)*				
Low	398 (51)	138 (54)	139 (53)	121 (47)
Intermediate	294 (38)	98 (38)	93 (35)	103 (40)
High	89 (11)	22 (9)	31 (12)	36 (14)
Lifestyle				
Smoking status, *n* (%)*				
Current	136 (17)	61 (24)	47 (18)	28 (11)
Former	360 (46)	112 (43)	124 (47)	124 (47)
Never	288 (37)	87 (34)	91 (35)	110 (42)
Physical activity, h/week*	18.4 [9.0–29.0]	15.5 [7.5–27.8]	19.2 [9.5–31.0]	19.0 [9.7–28.0]
Alcohol consumption, *n* (%)				
No consumption	142 (18)	46 (18)	50 (19)	46 (18)
0 – 13 glasses per week	482 (61)	151 (58)	164 (62)	167 (64)
≥ 14 glasses per week	165 (21)	65 (25)	50 (19)	50 (19)
BMI, kg/m^2^	27.7 ± 4.0	27.4 ± 3.9	27.8 ± 3.8	27.8 ± 4.3
Clinical characteristics				
Glucose status, *n* (%)				
Normal glucose metabolism	287 (36)	91 (35)	92 (35)	103 (39)
Impaired glucose metabolism	181 (23)	64 (25)	62 (24)	55 (21)
Type 2 diabetes mellitus	320 (41)	106 (41)	109 (41)	105 (40)
Prior CVD, *n *(%)*	417 (54)	152 (60)	133 (52)	132 (51)
Hypertension, *n* (%)	552 (70)	184 (70)	183 (70)	185 (70)
Systolic blood pressure, mmHg	142 ± 20	141 ± 19	143 ± 20	142 ± 22
HbA1c, mmol/mol	43.2 ± 8.5	42.3 ± 8.4	43.3 ± 8.1	43.5 ± 9.3
Triglycerides, mmol/l	1.4 [1.0–1.9]	1.4 [1.0–1.9]	1.4 [1.0–1.9]	1.4 [1.0–1.8]
Total cholesterol, mmol/l	5.7 ± 1.0	5.7 ± 1.0	5.7 ± 1.0	5.7 ± 1.1
LDL cholesterol, mmol/l	3.6 ± 0.9	3.5 ± 0.9	3.6 ± 0.9	3.7 ± 0.9
HDL cholesterol, mmol/l	1.4 ± 0.4	1.4 ± 0.4	1.4 ± 0.4	1.4 ± 0.4
CRP, mg/l*	2.0 [0.8–4.0]	2.1 [1.1–4.4]	2.1 [1.1–4.3]	1.6 [0.7–3.5]
eGFR, ml/min/1.73m^2^*	81 ± 14	79 ± 15	83 ± 12	82 ± 14
Use of PPIs, *n* (%)*	33 (4)	16 (6)	12 (5)	5 (2)
Dietary intake				
Energy intake, kcal/day	1936 ± 506	1961 ± 512	1857 ± 486	1987 ± 515
Dietary fibre, g/day*	23.3 ± 4.9	19.9 ± 4.0	23.2 ± 3.7	26.7 ± 4.5
Calcium intake, mg/day*	1062 ± 312	883 ± 229	1092 ± 273	1209 ± 336
Phosphorus, mg/day*	1439 ± 240	1248 ± 178	1469 ± 175	1596 ± 220
Vascular measurements				
Intima-media thickness, mm	0.86 ± 0.19	0.86 ± 0.163	0.86 ± 0.18	0.86 ± 0.17
Augmentation index, %	32.4 ± 8.7	32.5 ± 9.0	33.2 ± 8.7	31.5 ± 8.5
Peripheral arterial disease, *n* (%)	43 (7.1)	15 (7.5)	14 (7.3)	(14) 6.7
Pulse wave velocity, m/s	10.3 ± 5.2	10.4 ± 7.6	10.5 ± 3.3	10.2 ± 3.9
Flow-mediated dilatation, %	3.8 ± .9	3.7 ± 4.2	3.6 ± 3.4	4.0 ± 3.9

Normally distributed data are presented as mean ± standard deviation, non-normally distributed data as median [interquartile range], categorical data as %. *BMI* body mass index, *CVD* cardiovascular disease, *HbA1c* haemoglobin A1c, *LDL* low-density lipoprotein, *HDL* high-density lipoprotein, *CRP* C-reactive protein, *eGFR* estimated glomerular filtration rate (CKD-EPI 2009),* PPI* proton-pump inhibitor. Intima-media thickness *n* = 733, augmentation index *n* = 614, peripheral arterial disease (ankle brachial index < 0.9) *n* = 603, pulse wave, velocity *n* = 317, flow-mediated dilatation *n* = 635. *p trend < 0.05 **Dietary intake is adjusted for total energy intake using the residual method. Tertiles are sex-specific and, therefore, values may overlap

Mean follow-up duration was 7.5 ± 0.6 year. Participants with a vascular follow-up visit were significantly younger and healthier, with less frequent DM2 or a history of CVD, and they had a slightly higher baseline magnesium intake compared to those with baseline measurements only. These differences were also reflected in more favourable baseline values of vascular measures for participants with follow-up measurements (Supplementary Table 1). Energy-adjusted magnesium intake correlated well with phosphate and fibre intake (*r* = 0.64 and *r* = 0.62, respectively) and a weaker correlation was found for calcium (*r* = 0.44), all *p* < 0.01.

### Cross-sectional associations

Measurements of vascular structure and function at baseline did not differ significantly between the magnesium intake tertiles (Table 1**).** No crude or multivariable-adjusted cross-sectional associations were observed between magnesium intake tertiles and PWV, FMD, IMT, Aix or prevalent PAD (Table 2)**.** For FMD, results were also stratified according to the presence of CVD at baseline, because of a significant interaction between magnesium intake and prior CVD (*p* = 0.03). However, stratification did not considerably change these results (Supplementary Table 2). We found no effect modification by age, sex or glucose metabolism status for any of the vascular outcome measures (all *p* values  > 0.10).

**Table 2:** Cross-sectional association between sex-specific, energy-adjusted magnesium intake tertiles with intima-media thickness (*n* = 733), augmentation index (*n* = 614), pulse wave velocity (*n* = 317), flow-mediated dilatation (*n* = 635) and peripheral arterial disease (*n* = 589)

	Magnesium intake tertiles*			*p* trend
	Tertile 1	Tertile 2	Tertile 3	
Median magnesium intake, mg/day	282	328	371	
Range of magnesium intake (min–max), mg/day	136–313	303–350	345–530	
Beta (95% CI)				
IMT (mm)	*n* = 237	*n* = 248	*n* = 248	
Model 1	Ref. (0.0)	0.00 (−0.03, 0.03)	0.01 (−0.02, 0.04)	0.38
Model 2	Ref. (0.0)	0.00 (−0.03, 0.03)	0.01 (−0.02, 0.04)	0.38
Model 3	Ref. (0.0)	0.00 (−0.03, 0.03)	0.02 (−0.02, 0.05)	0.31
Aix (%)	*n* = 201	*n* = 200	*n* = 113	
Model 1	Ref. (0.0)	0.25 (−1.33, 1.82)	−0.97 (−2.53, 0.59)	0.22
Model 2	Ref. (0.0)	0.38 (−1.18, 1.94)	−0.49 (−2.05, 1.06)	0.53
Model 3	Ref. (0.0)	0.56 (−1.11, 2.23)	0.24 (−1.65, 2.14)	0.81
PWV (m/s)	*n* = 99	*n* = 111	*n* = 107	
Model 1	Ref. (0.0)	−0.05 (−1.44, 1.34)	0.08 (−1.33, 1.50)	0.91
Model 2	Ref. (0.0)	−0.07 (−1.40, 1.25)	0.13 (−1.23, 1.49)	0.85
Model 3	Ref. (0.0)	−0.31 (−1.79, 1.17)	−0.21 (−1.95, 1.52)	0.82
FMD (%)	*n* = 210	*n* = 206	*n* = 219	
Model 1	Ref. (0.0)	−0.08 (−0.80, 0.64)	0.10 (−0.61, 0.81)	0.78
Model 2	Ref. (0.0)	−0.12 (−0.83, 0.59)	−0.07 (−0.77, 0.63)	0.85
Model 3	Ref. (0.0)	−0.04 (−0.79, 0.70)	−0.03 (−0.89, 0.83)	0.94
OR (95% CI)				
ABI prevalent PAD	*n* = 201 *n* = 15	*n* = 192 *n* = 14	*n* = 196 *n* = 14	
Model 1	Ref. (1.0)	1.00 (0.46, 2.16)	1.02 (0.47, 2.21)	0.96
Model 2	Ref. (1.0)	1.19 (0.52, 2.71)	1.50 (0.64, 3.52)	0.36
Model 3	Ref. (1.0)	1.30 (0.54, 3.18)	1.75 (0.63, 4.88)	0.28

Model 1: age, sex and glucose status;

Model 2: model 1 + prior CVD, smoking status and systolic blood pressure;

Model 3: model 2 + caloric intake and energy-adjusted fibre intake

*ABI* ankle-brachial index, *Aix* augmentation index, *CI* confidence interval, *CVD* cardiovascular disease, *FMD* flow-mediated dilatation, *IMT* intima-media thickness, *Mg* magnesium, *OR* odds ratio, *PAD* peripheral artery disease (ABI  < 0.9), *PWV* pulse wave velocity, *Ref.* reference category

*Dietary intake is adjusted for total energy intake using the residual method. Tertiles are sex-specific and therefore magnesium intake values may overlap

### Longitudinal associations

Overall, both IMT and Aix increased significantly from a mean IMT of 0.84 ± 0.16 mm at baseline to 0.88 ± 0.16 mm at follow up, and a mean Aix of 31.4 ± 8.6% – 34.2 ± 8.4%, both *p* < 0.01. Absolute outcome and changes of IMT, Aix and incident PAD after 8 year of follow-up did not differ significantly across magnesium intake tertiles (Table 3)**.** After full adjustment, we did not observe associations between the magnesium intake tertiles and any of these vascular measurements (Table 4). We found no effect modification by age, sex, glucose metabolism status or prior CVD.

**Table 3:** Markers of vascular structure and function at baseline and after 8 years of follow-up for all participants with follow-up measurements of intimal media thickness, augmentation index and ankle-brachial index, according to sex-specific and energy-adjusted magnesium intake tertiles (*n* = 432)

	Magnesium intake tertiles*			*p* trend
	Tertile 1	Tertile 2	Tertile 3	
Median magnesium intake, mg/day	291	338	381	
Range of magnesium intake (min–max), mg/day	150–325	310–359	353–542	
IMT (mm)	*n* = 133	*n* = 136	*n* = 135	
Baseline	0.84 ± 0.14	0.84 ± 0.17	0.84 ± 0.17	0.80
Follow-up	0.87 ± 0.15	0.88 ± 0.15	0.88 ± 0.17	0.46
Delta IMT	0.04 ± 0.17	0.04 ± 0.18	0.04 ± 0.19	0.67
Aix (%)	*n* = 115	*n* = 107	*n* = 110	
Baseline	31.1 ± 8.8	32.5 ± 8.7	30.8 ± 8.3	0.78
Follow-up	33.1 ± 9.1	35.1 ± 7.7	34.6 ± 8.0	0.16
Delta Aix	2.0 ± 9.2	2.6 ± 9.2	3.9 ± 9.4	0.12
ABI	*n* = 116	*n* = 114	*n* = 115	
Prevalent PAD, *n* (%)	2 (1.7)	3 (2.6)	2 (1.7)	
Incident PAD**, *n* (%)	13/114 (11.4)	11/111 (9.9)	10/103 (8.8)	0.53

Data are presented as mean ± standard deviation, categorical data as n (%)

IMT *n* = 404, Aix *n* = 332, ABI *n* = 345

*ABI* Ankle-brachial index, *Aix* augmentation index, *IMT* intima-media thickness, *Mg* magnesium, *PAD* peripheral artery disease (ABI < 0.9)

*Dietary intake is adjusted for total energy intake using the residual method. Tertiles are sex-specific and, therefore, values may overlap

**The reported number of incident PAD are with the exclusion of prevalent PAD cases

**Table 4:** Longitudinal association between sex-specific, energy-adjusted magnesium intake tertiles with intima-media thickness (*n* = 415), augmentation index (*n* = 361) and the odds ratio of incident peripheral artery disease (*n* = 344) at 8 years of follow-up

	Magnesium intake tertiles*			*p* trend
	Tertile 1	Tertile 2	Tertile 3	
Median magnesium intake, mg/day	291	338	381	
Range of magnesium intake (min–max), mg/day	150–325	310–359	353–542	
Beta (95% CI)				
IMT (mm)	*n* = 137	*n* = 140	*n* = 138	
Model 1	Ref. (0.0)	0.01 (−0.03, 0.04)	0.02 (−0.02, 0.05)	0.37
Model 2	Ref. (0.0)	0.01 (−0.03, 0.04)	0.02 (−0.02, 0.05)	0.31
Model 3	Ref. (0.0)	0.00 (−0.04, 0.04)	0.01 (−0.03, 0.06)	0.57
Aix (%)	*n* = 122	*n* = 117	*n* = 122	
Model 1	Ref. (0.0)	1.59 (−0.40, 3.57)	1.76 (−0.20, 3.72)	0.08
Model 2	Ref. (0.0)	1.65 (−0.33, 3.63)	1.76 (−0.20, 3.72)	0.08
Model 3	Ref. (0.0)	1.04 (−1.06, 3.15)	0.70 (−1.69, 3.07)	0.56
OR (95% CI)				
ABI	*n* = 114	*n* = 114	*n* = 116	
Incident PAD	*n* = 13	*n* = 12	*n* = 12	
Model 1	Ref. (1.0)	0.67 (0.23, 1.92)	0.99 (0.34, 2.89)	0.92
Model 2	Ref. (1.0)	0.61 (0.20, 1.84)	0.94 (0.31, 2.85)	0.86
Model 3	Ref. (1.0)	0.59 (0.18, 1.88)	0.84 (0.23, 3.11)	0.77

*Dietary intake is adjusted for total energy intake using the residual method. Tertiles are sex-specific and, therefore, values may overlap

Model 1: age, sex and glucose status;

Model 2: model 1 + prior CVD, smoking status and systolic blood pressure;

Model 3: model 2 + caloric intake and energy adjusted fibre intake

*ABI* ankle-brachial index, *Aix* augmentation index, *CI* confidence interval, *IMT* intima-media thickness, *OR* odds ratio, *PAD* peripheral artery disease, *Ref.* reference category. The longitudinal associations are adjusted for time of follow-up and participants with PAD at baseline were excluded for the association with PAD (ABI < 0.9)

### Sensitivity analysis

Complete case analysis including, 757 and 417 participants for the cross-sectional and longitudinal cohorts, respectively, did not change the results for any of the vascular outcomes (Supplementary Table 3 and 4). The sensitivity analysis with absolute magnesium intake tertiles and energy-unadjusted intake variables did not considerably change the results (Supplementary Table 5 and 6). Although the cross-sectional energy-unadjusted analysis of Aix demonstrated a lower Aix for the highest compared to the lowest magnesium tertile, −1.71% (95% confidence interval −3.27, −0.14) and *p* trend < 0.01, adding fibre intake to the model erased this inverse association (Supplementary Table 5).

## Discussion

We found no evidence for cross-sectional or longitudinal associations between dietary magnesium intake and markers of vascular structure and function including IMT, Aix, PWV, FMD and PAD.

Our results confirm the very limited previous literature on magnesium intake and these vascular makers. In two large population-based cohorts, the Atherosclerosis Risk in Communities (ARIC) cohort and the Multi-Ethnic Study of Atherosclerosis (MESA) cohort, cross-sectional and multivariable associations for dietary magnesium and IMT were absent [29, 30]. In contrast, one smaller cohort study in healthy participants and one dietary intervention study in people with diabetes demonstrated age adjusted, inverse associations between (baseline) magnesium intake and IMT [31, 32]. However, these analyses were not energy- or fibre intake adjusted and also no association between magnesium intake and PWV was found [32]. To our knowledge, no studies have reported on dietary magnesium in relation to Aix, FMD or PAD. Other parameters of vascular structure and function such as coronary or aortic calcification, heart failure and blood pressure have been studied more frequently in relation to dietary magnesium, but these studies also revealed inconsistent results [5, 7, 10, 51–55]. Fibre or ‘diet’ was an imported confounder in most of these studies, often attenuating or erasing associations when adjusted for. Adjustment for fibre intake also considerably influenced most of our analyses of vascular outcomes, although these associations were not significant in crude or in fully-adjusted models. We also considered confounding by physical activity, alcohol intake and, education, however, this did not materially change the results (data not shown).

The absence of associations between magnesium intake and all markers of vascular structure and function in this cohort contradicts the more established association between dietary magnesium intake and CVD and mortality observed in systematic reviews and meta-analyses [3–7]. Similarly, these results are not in line with the mainly confirmative studies of serum magnesium concentrations in relation to vascular outcomes [16–20, 56, 57]. These inconsistencies could be explained in several ways. First of all, due to complex magnesium homeostasis—balancing fractional magnesium absorption, shifts between intracellular and extracellular compartments, uptake and release form the bone and renal excretion—it is possible that dietary magnesium intake has no or too little effect on serum or total body magnesium and explain why no associations between magnesium intake and vascular parameters are found. Secondly, our sample size could be too small (lack of power) to demonstrate an association, since most confirmative studies were larger cohorts or meta-analyses. Furthermore, not all studies that found an inverse association between magnesium intake and (other) vascular markers adjusted for energy intake or dietary intake of other nutrients such as fibre and, therefore, possibly incorrectly attributed this association to magnesium intake, while in fact this may reflect a healthier diet or better nutritional status. Another explanation is the relatively high percentage of comorbidities of this vascular screening sub-cohort, including prior CVD (55%), hypertension (70%), DM2 (41%) and IGT (23%) and already unfavourable vascular outcomes at baseline. Based on the understanding that magnesium inhibits vascular calcification and inflammation rather than reversing established vascular impairment [58], we cannot exclude that the assumed beneficial effect of higher magnesium intake is too small to be detected in this already severely vascular affected high-risk population. In line, a recent sub-analysis of the ARIC cohort did not show an association between serum magnesium and PAD risk in high-risk individuals, while this association was present in the overall population-based cohort [59]. Lastly, the range of magnesium intake within a regular diet is relatively small. The absolute magnesium intake of 321 mg/day (IQR of 273–375) within this cohort, is close to the European Food Safety Authority magnesium intake recommendation of 300 mg and 350 mg per day for adult women and men, respectively. Also the absolute dietary magnesium intake range of 109–701 mg/day is close to the intake range of 96–425 mg/day described in a systematic review and meta-analysis of multiple prospective cohort studies on magnesium intake [10]. However, this dietary range may be too small to detect substantial and consistent associations on vascular parameters, hardly influencing serum magnesium levels. Magnesium intervention studies, mostly doubling daily magnesium intake (up to 610 mg of elemental magnesium a day on top of dietary intake) are less vulnerable for confounding by dietary components accompanying a higher magnesium diet and are more likely to positively influence magnesium balance. Indeed, several-magnesium supplementation studies within comparable or even higher risk study populations did find an effect of magnesium supplementation on IMT, PWV and FMD [24–27, 60–62].

### Strengths and limitations

The strengths of this study include its combination of a comprehensive set of markers of vascular structure and function in a single, cohort and its prospective design. In addition, the selection of a high-risk population with already unfavourable vascular outcomes at baseline is one of the strengths, since results of dietary exposure are generally most pronounced for those at high risk. To our knowledge, it is the first study that investigated dietary magnesium intake in relation to FMD, PAD and Aix. We were able to adjust for a variety of covariates, including energy and fibre intake and we could study the effect modification for glucose status, sex, age and a history of CVD.

A limitation of our study is the sample size that could have been insufficient to detect significant and clinically relevant differences for some of the vascular markers, potentially in the case of PWV [63] and for the risk of PAD. However, for IMT, FMD and Aix, the effect estimates and 95% CI did not include clinical relevant differences [64], and, therefore, these null results are not due to lack of power but rather reflect very small differences. Another limitations is that assessment of magnesium intake with the FFQ was not validated to estimate magnesium intake. However, the FFQ was validated for other nutrients and correlated well with fibre intake and adequately ranks subjects according to intake of most food groups, energy and fibre intake [35, 36]. Therefore, we assume that the FFQ correctly ranks subjects according to their magnesium intake. With only estimated magnesium intake at baseline, possible dietary changes affecting magnesium intake during follow-up are not taken into account in the longitudinal association, though we can assume that dietary intake is relatively stable over the years [65]. Unfortunately, we only had follow-up measurements of three out of five vascular outcomes and although the majority of participants had measurements of all concerning vascular outcomes, missing data in outcome measurements could be due to measurement specific or participant-related complications, or simply because of separate visits for some vascular measurements. In addition, the longitudinal associations may be biased due to a selective and high rate of lost to follow-up (attrition bias). However, we observed similar results for the cross-sectional analyses of concerning outcome parameters. Every vascular measurement has its own limitations, but generally they are complex to perform and these measurements are subject to inter-individual variation and variation due to external factors, such as temperature, medicine or caffeine intake. Finally, inherent to our studies observational design, there may be residual confounding due to unmeasured factors. However, considering the overall absence of associations between magnesium intake and the vascular markers, residual confounding as well as adjustment for multiple testing would not change the results.

### Conclusions and future research

Dietary intake of magnesium is not associated with vascular structure and function within this prospective cohort. It is unknown if a substantial higher magnesium intake in the general population may be beneficial for vascular outcome parameters. Therefore, we suggest future studies to focus on magnesium supplementation on top of dietary magnesium intake in relation to vascular outcomes, especially in a long-term and preventive setting.

## Supplementary Information

Below is the link to the electronic supplementary material.

Supplementary file1 (DOCX 37 KB)
