# Supplementary material for: Magnesium intake and vascular structure and function: the Hoorn Study
Source: Eur J Nutr. 2021 Sep 7;61(2):653–64. doi: 10.1007/s00394-021-02667-0 (PMC8854245; doi:10.1007/s00394-021-02667-0)
Supplement: Supplementary file 1 — Supplementary file1 (DOCX 37 KB) [file 394_2021_2667_MOESM1_ESM.docx]

**Supplementary Table 1**. Baseline characteristics of the vascular screening sub-cohort of the Hoorn study according to presence of follow-up visit (n=789).

|  | Participants **without** follow-up measurement | Participants **with**  follow-up measurement | *P*-value |
| --- | --- | --- | --- |
| n | 357 | 432 |  |
| ***Demographics*** |  |  |  |
| Age, years | 70.9±7.2 | 66.4±6.6 | <0.01 |
| Men, n (%) | 178(50) | 223(50) | 0.62 |
| Education level, n (%) |  |  | <0.01 |
| Low | 206(59) | 192(45) |  |
| Intermediate | 114(32) | 180(42) |  |
| High | 32(9) | 57(13) |  |
| ***Lifestyle*** |  |  |  |
| Smoking status, n (%) |  |  | 0.02 |
| Current | 76(22) | 60(14) |  |
| Former | 160(45) | 200(47) |  |
| Never | 118(33) | 170(40) |  |
| Physical activity, h/week | 17.0[7.0 29.7] | 18.5[10.5-28.0] | 0.12 |
| Alcohol consumption, n (%) |  |  | <0.01 |
| No consumption | 84(24) | 58(13) |  |
| 0 – 13 glasses per week | 203(57) | 279(65) |  |
| ≥14 glasses per week | 70(20) | 95(22) |  |
| BMI, kg/m^2^ | 27.8±4.4 | 27.6±3.6 | 0.57 |
| ***Clinical characteristics*** |  |  |  |
| Glucose status, n (%) |  |  | <0.01 |
| Normal glucose tolerance | 99(28) | 187(43) |  |
| Impaired glucose tolerance | 83(23) | 98(23) |  |
| Type 2 diabetes mellitus | 175(49) | 145(33) |  |
| Prior cardiovascular disease, n (%) | 211(61) | 206(49) | <0.01 |
| Hypertension, n (%) | 277(78) | 275(64) | <0.01 |
| Systolic blood pressure, mmHg | 145±21 | 139±19 | <0.01 |
| HbA1c, mmol/mol | 44.3±8.7 | 42.5±8.5 | <0.01 |
| Triglycerides, mmol/l | 1.4[1.1-1.9] | 1.3[1.0-1.8] | 0.07 |
| Total cholesterol, mmol/l | 5.6±1.0 | 5.7±1.0 | 0.40 |
| LDL cholesterol, mmol/l | 3.6±0.9 | 3.6±0.9 | 0.40 |
| HDL- cholesterol, mmol/l | 1.4±0.4 | 1.4±0.4 | 0.15 |
| CRP, mg/l | 2.2[1.2-4.8] | 1.7[0.8–3.4] | <0.01 |
| eGFR, ml/min/1.73m^2^ | 80±15 | 83±12 | 0.01 |
| Use of PPI, n (%) | 13(4) | 20(5) | 0.59 |
| ***Dietary intake*** |  |  |  |
| Energy intake, kcal/day | 1865±484 | 1994±518 | <0.01 |
| Magnesium intake, mg/day* | 324±46 | 331±51 | 0.05 |
| Dietary fibre, g/day* | 24±5 | 24±5 | 0.85 |
| Calcium intake, mg/day* | 1064±321 | 1060±306 | 0.85 |
| Phosphorus, mg/day* | 1434 ± 242 | 1443 ± 238 | 0.58 |
| ***Vascular measurements*** |  |  |  |
| Intima media thickness, mm | 0.89±0.18 | 0.84±0.16 | <0.01 |
| Augmentation index, % | 33.6±8.9 | 31.5±8,5 | <0.01 |
| Peripheral arterial disease, n (%) | 29(12) | 14(3) | <0.01 |
| Pulse wave velocity, m/s | 11.0±4.7 | 10.0±5.5 | 0.10 |
| Flow mediated dilatation, % | 3.2±4.4 | 4.2±3.4 | <0.01 |

Normally distributed data are presented as mean ± standard deviation, non-normally distributed
data as median [interquartile range], categorical data as %. Abbreviations: BMI, Body Mass Index; HbA1c, Haemoglobin A1c; LDL, low-density lipoprotein; HDL, high-density lipoprotein; CRP, C-reactive protein; eGFR, estimated Glomerular Filtration Rate (CKD-EPI 2009); PPI, proton-pump inhibitor. Peripheral arterial disease is defined as a left and/or right ankle-brachial index <0.9*.**Dietary intake variables were adjusted for total energy intake using the residual method.

**Supplementary Table 2.** Cross-sectional association between sex-specific, energy-adjusted magnesium intake tertiles and FMD (n=635), stratified according to prior CVD.

|  | **Magnesium intake tertiles*** | | |  |
| --- | --- | --- | --- | --- |
|  | Tertile 1 | Tertile 2 | Tertile 3 | *P*-trend |
| Median magnesium intake, mg/day | 282 | 328 | 371 |  |
| Range of magnesium intake (min-max), mg/day | 136-313 | 303-350 | 345-530 |  |
| **FMD (%)** | | | | |
| *NO prior CVD* | n=87 | n=99 | n=110 |  |
| Model 1 | Ref. (0.0) | 0.42(-0.57,1.41) | 0.81(-0.15,1.78) | 0.10 |
| Model 2 | Ref. (0.0) | 0.34(-0.64,1.31) | 0.59(-0.38,1.55) | 0.23 |
| Model 3 | Ref. (0.0) | 0.42(-0.61,1.46) | 0.63(-0.55,1.80) | 0.30 |
| *Prior CVD* | n=123 | n=107 | n=109 |  |
| Model 1 | Ref. (0.0) | -0.54(-1.59,0.51) | -0.64(-1.68,0.40) | 0.22 |
| Model 2 | Ref. (0.0) | -0.46(-1.50,0.59) | -0.67(-1.72,0.37) | 0.20 |
| Model 3 | Ref. (0.0) | -0.39(-1.48,0.70) | -0.62(-1.88,0.64) | 0.33 |

CVD, cardiovascular disease; FMD, flow-mediated dilatation; Mg, magnesium; Ref., reference category. *Dietary intake is adjusted for total energy intake using the residual method. Interaction term prior CVD *P*=0.03. Effect sizes are beta’s (95% confidence intervals)

Model 1: age, sex and glucose status;
Model 2: model 1 + smoking status and systolic blood pressure;
Model 3: model 2 + caloric intake and energy adjusted fibre intake.

**Supplementary Table 3.** Full-case analysis for the cross-sectional association between sex-specific, energy-adjusted magnesium intake tertiles with PWV (n=310) and FMD (n=608), IMT (n=701), Aix (n=601) and PAD (n=588).

|  | **Magnesium intake tertiles*** | | |  |
| --- | --- | --- | --- | --- |
|  | Tertile 1 | Tertile 2 | Tertile 3 | *P*-trend |
| Median magnesium intake, mg/day | 282 | 328 | 371 | |
| Range of magnesium intake  (min-max), mg/day | 136-313 | 303-350 | 345-530 | |
|  | Beta (95%CI) | | |  |
| **IMT (mm)** | n=226 | n=233 | n=242 |  |
| Model 1 | Ref. (0.0) | 0.00(-0.03,0.03) | 0.01(-0.01,0.04) | 0.31 |
| Model 2 | Ref. (0.0) | 0.00(-0.03,0.03) | 0.02(-0.01,0.05) | 0.27 |
| Model 3 | Ref. (0.0) | 0.00(-0.03,0.04) | 0.02(-0.01,0.06) | 0.23 |
| **Aix (%)** | n=195 | n=195 | n=211 |  |
| Model 1 | Ref. (0.0) | 0.46(-1.13,2.06) | -0.84(-2.41,0.73) | 0.29 |
| Model 2 | Ref. (0.0) | 0.61(-0.97,2.19) | -0.39(-1.95,1.18) | 0.62 |
| Model 3 | Ref. (0.0) | 0.87(-0.81,2.56) | 0.51(-1.40,2.42) | 0.61 |
| **PWV (m/s)** | n=97 | n=106 | n=107 |  |
| Model 1 | Ref. (0.0) | -0.18(-1.60,1.24) | 0.05(-1.33,1.50) | 0.94 |
| Model 2 | Ref. (0.0) | -0.13(-1.48,1.23) | 0.15(-1.22,1.53) | 0.82 |
| Model 3 | Ref. (0.0) | -0.40(-1.90,1.11) | -0.26(-2.02,1.50) | 0.79 |
| **FMD (%)** | n=201 | n=194 | n=213 |  |
| Model 1 | Ref. (0.0) | -0.08(-0.81,0.66) | 0.16(-0.56,0.88) | 0.66 |
| Model 2 | Ref. (0.0) | -0.12(-0.85,0.61) | -0.01(-0.73,0.71) | 0.98 |
| Model 3 | Ref. (0.0) | -0.05(-0.81,0.72) | -0.04(-0.84,0.92) | 0.93 |
|  | OR (95%CI) | | |  |
| **ABI**  Prevalent PAD | n=195 n=15 | n=187 n=14 | n=206  n=14 |  |
| Model 1 | Ref. (1.0) | 1.01(0.47,2.19) | 1.02(0.47,2.24) | 0.94 |
| Model 2 | Ref. (1.0) | 1.20(0.53,2.76) | 1.55(0.66,3.65) | 0.32 |
| Model 3 | Ref. (1.0) | 1.32(0.54,3.23) | 1.81(0.65,5.04) | 0.26 |

ABI, ankle-brachial index; Aix, augmentation index; CI, confidence interval; CVD, cardiovascular disease; FMD, flow-mediated dilatation; IMT, intima-media thickness; Mg, magnesium; OR, odds ratio; PAD, peripheral artery disease (ABI < 0.9); PWV, pulse wave velocity; Ref., reference category.*Dietary intake is adjusted for total energy intake using the residual method. Tertiles are sex-specific and, therefore, values may overlap.

Model 1: age, sex and glucose status;
Model 2: model 1 + prior CVD, smoking status and systolic blood pressure;
Model 3: model 2 + caloric intake and energy adjusted fibre intake.

**Supplementary Table 4**. Full-case analysis for the longitudinal association between sex-specific, energy-adjusted magnesium intake tertiles with intima-media thickness (n=389), augmentation index (n=328) and peripheral artery disease (n=329) at 8 years of follow-up.

|  | **Magnesium intake tertiles*** | | |  |
| --- | --- | --- | --- | --- |
|  | **Tertile 1** | **Tertile 2** | **Tertile 3** | *P*-trend |
| Median magnesium intake, mg/day  Range of magnesium intake  (min-max), mg/day | 291  150-325 | 338  310-359 | 381  353-542 |  |
|  | Beta (95%CI) | | |  |
| **IMT (mm)** | n=128 | n=130 | n=131 |  |
| Model 1 | Ref. (0.0) | 0.01(-0.03,0.05) | 0.02(-0.02,0.06) | 0.28 |
| Model 2 | Ref. (0.0) | 0.01(-0.03,0.05) | 0.02(-0.01,0.05) | 0.22 |
| Model 3 | Ref. (0.0) | 0.00(-0.03,0.04) | 0.02(-0.03,0.06) | 0.48 |
| **Aix (%)** | n=112 | n=106 | n=110 |  |
| Model 1 | Ref. (0.0) | 1.36(-0.65,3.38) | 1.43 (-0.57,3.43) | 0.15 |
| Model 2 | Ref. (0.0) | 1.39(-0.62,3.40) | 1.49(-0.52,3.50) | 0.14 |
| Model 3 | Ref. (0.0) | 0.74 (-1.39,2.87) | 0.34(-2.08,2.76) | 0.77 |
|  | OR (95%CI) | | |  |
| **ABI**  Incident PAD | n=110 n=12 | n=108 n=11 | n=111  n=9 |  |
| Model 1 | Ref. (1.0) | 0.67(0.23,2.01) | 1.02(0.34,3.10) | 0.96 |
| Model 2 | Ref. (1.0) | 0.64(0.21,1.97) | 0.94(0.32,3.14) | 0.94 |
| Model 3 | Ref. (1.0) | 0.62(0.19,2.07) | 0.91(0.23,3.58) | 0.86 |

ABI, ankle-brachial index; Aix, augmentation index; CI, confidence interval; IMT, intima-media thickness; Mg, magnesium; OR, odds ratio; PAD, peripheral artery disease. Ref., reference category. The longitudinal associations are adjusted for time of follow-up and participants with PAD at baseline were excluded for the association with PAD (ABI < 0.9). *Dietary intake is adjusted for total energy intake using the residual method. Tertiles are sex-specific and, therefore, values may overlap.

Model 1: age, sex and glucose status;

Model 2: model 1 + prior CVD, smoking status and systolic blood pressure;

Model 3: model 2 + caloric intake and energy adjusted fibre intake.

**Supplementary Table 5.** Cross-sectional association between sex-specific, *absolute* magnesium intake tertiles with PWV (n=317) and FMD (n=635), IMT (n=733), Aix (n=614) and PAD (n=603).

|  | Magnesium intake tertiles* | | |  |
| --- | --- | --- | --- | --- |
|  | Tertile 1 | Tertile 2 | Tertile 3 | *P*-trend |
| Median magnesium intake, mg/day | 252 | 321 | 397 |  |
| Range of magnesium intake  (min-max), mg/day | 109-314 | 271-373 | 328-701 |  |
|  | Beta (95% CI) | | |  |
| **IMT (mm)** | n=236 | n=245 | n=252 |  |
| Model 1 | Ref. (0.0) | 0.00(-0.03,0.03) | 0.00(-0.03,0.03) | 0.88 |
| Model 2 | Ref. (0.0) | 0.00(-0.02,0.03) | 0.00(-0.02,0.03) | 0.77 |
| Model 3 | Ref. (0.0) | 0.01(-0.02,0.04) | 0.02(-0.03,0.06) | 0.50 |
| **Aix (%)** | n=205 | n=208 | n=201 |  |
| Model 1 | Ref. (0.0) | -0.95(-2.50,0.60) | -2.02(-3.60,-0.44) | 0.01 |
| Model 2 | Ref. (0.0) | -0.69(-2.22,0.84) | -1.71(-3.27,-0.14) | <0.01 |
| Model 3 | Ref. (0.0) | -0.19(-1.91,1.54) | -0.54(-2.96,1.87) | 0.67 |
| **PWV (m/s)** | n=99 | n=111 | n=107 |  |
| Model 1 | Ref. (0.0) | -0.36(-1.76,1.05) | -0.09(-1.49,1.50) | 0.91 |
| Model 2 | Ref. (0.0) | -0.41(-1.74,0.93) | -0.03(-1.36,1.31) | 0.98 |
| Model 3 | Ref. (0.0) | -0.43(-1.97,1.11) | -0.05(-2.01,2.10) | 0.97 |
| **FMD (%)** | n=201 | n=214 | n=220 |  |
| Model 1 | Ref. (0.0) | 0.34(-0.38,1.06) | 0.39(-0.34,1.11) | 0.30 |
| Model 2 | Ref. (0.0) | 0.27(-0.44,0.98) | 0.25 (-0.46,0.97) | 0.43 |
| Model 3 | Ref. (0.0) | -0.07(-0.87,0.73) | -0.55(-1.64,0.54) | 0.43 |
|  | OR (95% CI) | | |  |
| **ABI**  Prevalent PAD | n=203 n=16 | n=204 n=15 | n=196  n=12 |  |
| Model 1 | Ref. (1.0) | 1.06(0.50,2.24) | 0.97(0.44,2.15) | 0.95 |
| Model 2 | Ref. (1.0) | 1.40(0.63,3.11 | 1.31(0.55,3.12) | 0.50 |
| Model 3 | Ref. (1.0) | 1.57(0.63,3.93) | 1.77(0.44,7.14) | 0.37 |

ABI, ankle-brachial index; Aix, augmentation index; CI, confidence interval; CVD, cardiovascular disease; FMD, flow-mediated dilatation; IMT, intima-media thickness; Mg, magnesium; OR, odds ratio; PAD, peripheral artery disease (ABI < 0.9); PWV, pulse wave velocity; Ref., reference category. *Tertiles are sex-specific and, therefore, values may overlap.

Model 1: age, sex and glucose status;
Model 2: model 1 + prior CVD, smoking status and systolic blood pressure;
Model 3: model 2 + caloric intake and energy adjusted fibre intake.

**Supplementary Table 6.** Longitudinal association between sex-specific, *absolute* magnesium intake tertiles with intima-media thickness (n=415), augmentation index (n=361), peripheral arterial disease (n=329) at 8 years of follow-up.

|  | Magnesium intake tertiles* | | |  |
| --- | --- | --- | --- | --- |
|  | Tertile 1 | Tertile 2 | Tertile 3 | *P*-trend |
| Median magnesium intake, mg/day | 263 | 326 | 420 |  |
| Range of magnesium intake  (min-max), mg/day | 159-319 | 277-388 | 332-700 |  |
|  | Beta (95% CI) | | |  |
| **IMT (mm)** | n=137 | n=136 | n=142 |  |
| Model 1 | Ref. (0.0) | 0.02(-0.02,0.06) | 0.00(-0.04,0.04) | 0.98 |
| Model 2 | Ref. (0.0) | 0.02(-0.02,0.06) | 0.00(-0.04,0.04) | 0.97 |
| Model 3 | Ref. (0.0) | 0.02(-0.02,0.06) | 0.00(-0.05,0.06) | 0.81 |
| **Aix (%)** | n=125 | n=122 | n=114 |  |
| Model 1 | Ref. (0.0) | 0.55(-1.42,2.52) | 1.15(-0.97,3.26) | 0.29 |
| Model 2 | Ref. (0.0) | 0.58(-1.38,2.55) | 1.17(-0.94,3.28) | 0.28 |
| Model 3 | Ref. (0.0) | 0.52(-1.69,2.73) | 1.60(-1.69,4.88) | 0.36 |
|  | OR (95% CI) | | |  |
| **ABI**  Incident PAD | n=114 n=10 | n=122 n=14 | n=108  n=6 |  |
| Model 1 | Ref. (1.0) | 2.71(0.94,7.79) | 0.88(0.24,3.25) | 0.90 |
| Model 2 | Ref. (1.0) | 2.87(0.98,8.37) | 0.88(0.24,3.27) | 0.89 |
| Model 3 | Ref. (1.0) | 2.29(0.70,7.44) | 0.40(0.06,2.77) | 0.71 |

ABI, ankle-brachial index; Aix, augmentation index; CI, confidence interval; IMT, intima-media thickness; Mg, magnesium; OR, odds ratio; PAD, peripheral artery disease. Ref., reference category. The longitudinal associations are adjusted for time of follow-up and participants with PAD at baseline were excluded for the association with PAD (ABI < 0.9). *Tertiles are sex-specific and, therefore, values may overlap.

Model 1: age, sex and glucose status;

Model 2: model 1 + prior CVD, smoking status and systolic blood pressure;

Model 3: model 2 + caloric intake and energy adjusted fibre intake.
